# Removing and reimplanting deep brain stimulation therapy devices in resistant OCD (when the patient does not respond): case report

**DOI:** 10.1186/s12888-016-0730-z

**Published:** 2016-02-06

**Authors:** Eva Real, Gerard Plans, Pino Alonso, Marco A. Aparicio, Cinto Segalàs, Narcís Cardoner, Carles Soriano-Mas, Clara López-Solà, José M. Menchón

**Affiliations:** Psychiatry Department, Bellvitge University Hospital, Bellvitge Biomedical Research Institute (IDIBELL), C/ Feixa Llarga s/n, 08907 Hospitalet del Llobregat, Barcelona, Spain; Carlos III Health Institute, Centro de Investigación Biomédica en Red de Salud Mental (CIBERSAM), Madrid, Spain; Department of Neurosurgery, Bellvitge University Hospital, C/ Feixa Llarga s/n, 08907 Hospitalet del Llobregat, Barcelona, Spain; Department of Clinical Sciences, School of Medicine, University of Barcelona, Barcelona, Spain; Mental Health Department, Corporació Sanitària Parc Taulí, Sabadell, Spain

**Keywords:** Deep brain stimulation, Obsessive-compulsive disorder, Device removal, Treatment outcome

## Abstract

**Background:**

Deep brain stimulation (DBS) is emerging as a promising tool in the treatment of refractory obsessive-compulsive disorder (OCD) but the search for the best target still continues. This issue is especially relevant when particularly resistant profiles are observed in some patients, which have been ascribed to individual responses to DBS according to differential patterns of connectivity. As patients have been implanted, new dilemmas have emerged, such as what to do when the patient does not respond to surgery.

**Case presentation:**

Here we describe a 22-year-old male with extremely severe OCD who did not respond to treatment with DBS in the nucleus accumbens, but who did respond after explanting and reimplanting leads targeting the ventral capsule-ventral striatum region. Information regarding the position of the electrodes for both surgeries is provided and possible brain structures affected during stimulation are reviewed. To our knowledge this case is the first in the literature reporting the removal and reimplantation of DBS leads for therapeutical benefits in a patient affected by a mental disorder.

**Conclusion:**

The capability for explantation and reimplantation of leads should be considered as part of the DBS therapy reversibility profile in resistant mental disorders, as it allows application in cases of non-response to the first surgery.

## Background

The scientific and ethical issues in the application of deep brain stimulation (DBS) in resistant psychiatric disorders continue to be debated (reviewed in [1]). In the case of obsessive-compulsive disorder (OCD), ablative psychosurgery has been the last-resort treatment for refractory patients in recent decades [2]. However, the therapeutic benefits reported for this approach are clouded by its potential adverse effects, especially impairment in frontal lobe functioning [3, 4]. In this context, DBS has emerged as an attractive alternative and has progressively replaced ablative surgery in resistant OCD and depression.

As patients have been implanted, new dilemmas have emerged, such as what to do when the patient does not respond to surgery. Device removal is often required for clinical cure when leads or generator pocket infection occurs. This procedure (and device reimplantation) has been previously reported for movement disorders [5–7], but not for therapeutical purposes in mental disorders. The patient described here is a treatment-refractory OCD participant on a DBS treatment program run by at the OCD Clinical and Research Unit of Bellvitge University Hospital. He was explanted and reimplanted (in a different location and using different leads) due to very low therapeutic benefit after the first surgery.

## Case presentation

### Clinical history

A 22-year-old male with extremely severe OCD underwent DBS and pulse generator implantation in September 2009. The patient and his family reported severe obsessive-compulsive symptomatology since childhood: at 11 he began to manifest magical thoughts and compulsions involving “just-right” symptoms, symmetry and perfectionism, slowness in writing, the need to hoard and to touch objects, and repeating and arranging rituals. The disease course had been progressively debilitating, with highly compulsive time wasting behavior that led to the abandonment of education and successive hospital admissions from the age of 13. After trying a wide variety of treatments the patient was enrolled on the DBS program. For reasons of space, some features of the case (as well as treatment resistance profile) are summarized in the Table 1.

**Table 1:** Patient characteristics and stimulation settings

Sex	Male			
Age at OCD Onset	11			
Age at surgery	19			
Worst YBOCS ever	38			
Worst HDRS ever	12			
Pharmacological resistance profile^1^	Fluoxetine 60 mg/d Fluvoxamine 300 mg/d Sertraline 200 mg/d Escitalopram 20 mg/d Clomipramine 300 mg/d		Venlafaxxine 150 mg/d Phenelzine 60 mg/d Pimozide 2 mg/d Risperidone 3 mg/d Amisulpride 200 mg/d Aripiprazole 15 mg/d	
	1st Surgery		2nd Surgery	
Treatment^2^	CLM 262.5 mg/d + ESCIT 20 mg/d + RIS 1.5 mg/d + AGOM 25 mg/d		CLM 265.5 mg/d + ESCIT 40 mg/d + RIS 1.5 mg/d	
Stimulation settings	0 + 1-, 210 μs, 135 Hz, 4 V	4 + 5-, 210 μs, 135 Hz, 4 V	0-1+, 270 μs, 60 Hz, 3 V	5-6+, 270 μs, 60 Hz, 3 V
Best YBOCS reached	35		26	
Best HDRS reached	12		7	
Best GAF reached	30		65	
Position of the center of the stimulation*	Left (0–1)	Right (4–5)	Left (0–1)	Right (5–6)
x	5,29	10,54	9,02	8,57
y	5,47	3,54	−3,12	−4,46
z	−4,26	−0,85	2,87	−1,32

^1^Pharmacological resistance profile refers to all the different pharmacological trials conducted during the whole follow-up (since the patient was first treated at 11 years old until he performed the first surgery). Doses of these drugs were maintained for at least 12 weeks, to complete the trials

^2^Treatment refers to the precise combination of drugs that the patient was taking when the best Y-BOCS and HDRS scores were achieved

*Distance from the anterior commissure (AC)

*Abbreviations*: *OCD* obsessive-compulsive disorder, *Y-BOCS* Yale-Brown Obsessive Compulsive Scale, *HDRS* Hamilton Depression Rating Scale, *GAF* Global Assessment Functioning scale, *CLM* clomipramine, *ESCIT* escitalopram, *RIS* risperidone, *AGOM* agomelatine, *x* lateral, *y* anteroposterior, *z* axial

### First DBS surgery

Surgical procedure was performed according to Bellvitge Hospital DBS protocol for resistant OCD (L.D. B-29406-2012). The study protocol was approved by the ethics committee of Bellvitge University Hospital (CEIC Ciutat Sanitària i Universitària de Bellvitge). Two quadripolar DBS electrodes (Model 3389 DBS lead; Medtronic, MN, USA) were bilaterally implanted in the nucleus accumbens (NAcc) region with the aid of a Leksell stereotactic frame (Elekta, Sweden). NAcc was one of the targets reported to be effective in the treatment of OCD with DBS [8, 9] and 3389 electrode was the one available in our setting at that moment. The target was referenced to the anterior commissure (AC) as described in previous studies [9]. The stereotactic brain surgery software BrainLab iPlan Stereotaxy 1.1 (Brain Lab, Germany) was used for target selection and trajectory planning. A postoperative stereotactic cranial CT scan was co-registered with the preoperative MRI to verify the final position of the electrodes within the first 24 hours. The position of each contact was referenced to the AC and coordinates were transferred to an anatomic atlas [10] to identify anatomic structure influenced during stimulation. The final position of the active contacts with respect to AC is shown in the Table 1.

Even though parameter testing and adjustment were the same as in other DBS implanted patients, no significant clinical improvements were achieved with the first surgery (Y-BOCS reduction from 38 to 35, less than 10 % of basal YBOCS). The patient’s quality of life remained very poor, and he needed further hospital admissions after DBS surgery. After a full explanation and with the consent of the patient and his family, the DBS electrodes were removed in March 2011. In April 2011 the patient underwent surgery for the second time. A baseline-assessment between the two implantations was done and clinical and psychometric measurements were performed (i.e.: Y-BOCS ratings dropped to the first intervention’s previous levels).

### Second DBS surgery

**Fig. 1:**
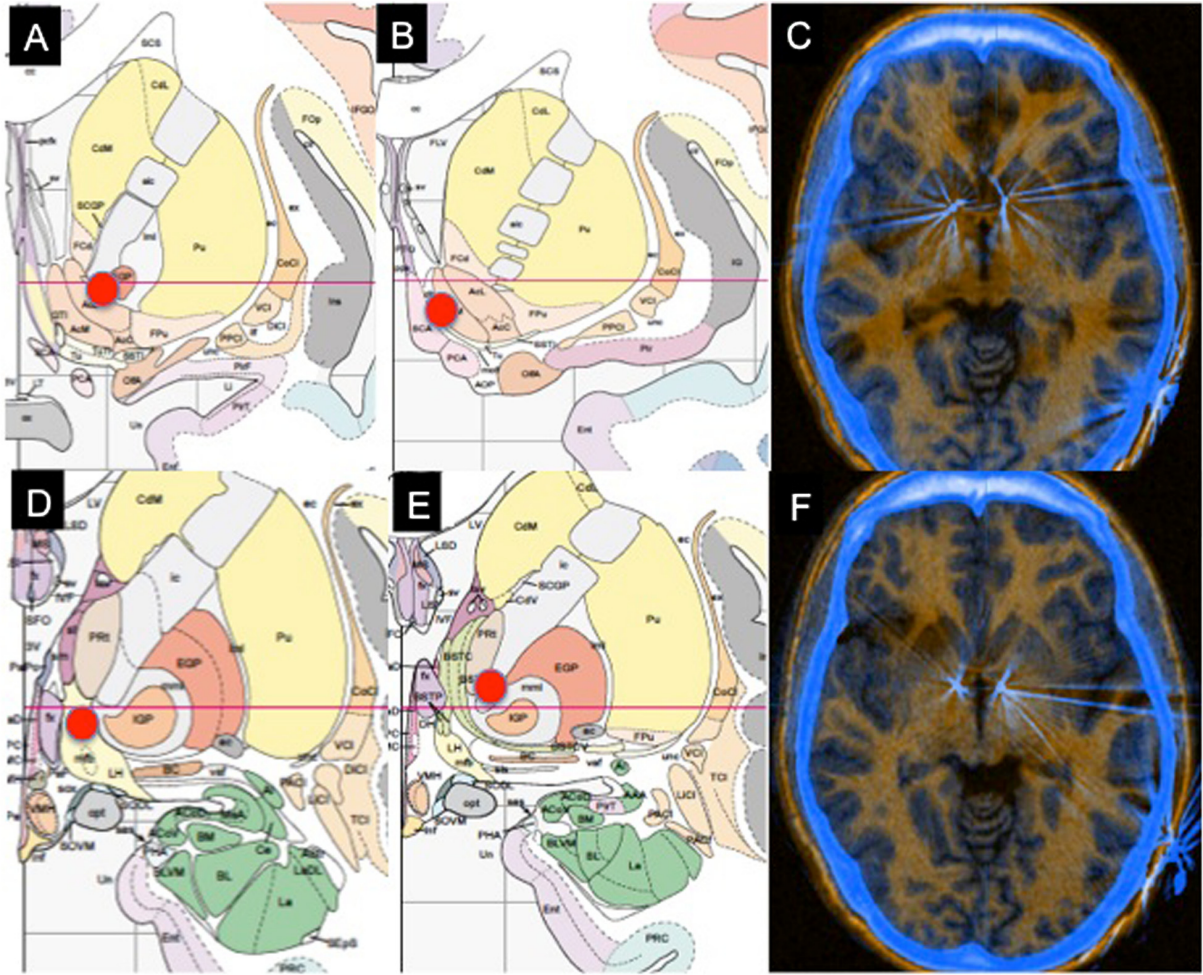
Anatomical location of the stimulation points and postoperative location of the electrodes on both surgeries. Red dots (A,B,D,E) show the position of the center of the stimulation after the first and second surgery superimposed in Mai atlas*†. **a** Coronal section 4,2 mm anterior to AC showing the middle point between the two active contacts on the right side after the first surgery. **b** Coronal section 5,8 mm anterior to AC showing the middle point between the two active contacts on the left side after the first surgery. **c** Postoperative CT fused with MRI showing the radiological position of the electrodes with respect to anterior comissure after the first surgery. **d** Coronal section 4,0 mm posterior to AC showing the middle point between the two active contacts on the right side after the second surgery. **e** Coronal section 2,7 mm posterior to AC showing the middle point between the two active contacts on the left side after the second surgery. **f** Postoperative CT fused with MRI showing radiological position of the electrodes with respect to anterior comissure after the second surgery. * Images on both sides correspond to frontal sections of left hemispheres of the human brain in Mai atlas. Frontal sections most closely related with anatomical location of the stimulation point have been chosen for the figure. † Elsevier Ltd. granted written permission to use, adapt and publish the images belonging to Atlas of the Human Brain, 3° ed (ISBN 9780123736031), Mai et al.

For the second surgical procedure we decided to use the ventral capsule/ventral striatum (VC/VS) target as it was reported to have the strongest evidence for therapeutic benefits at that moment [11]. The final theoretical target was calculated to be at the posterior border of the AC at the intercommissural level and 5 mm lateral to midline. The final trajectory was planned to cover the whole length of the anterior limb of the internal capsule (ALIC). For this purpose a Model 3391 DBS lead was used (the aim was to increase the setting options of the electric field, mainly in size, seeking the highest potential for clinical benefit in this especially resistant patient). The deepest active contacts on both sides (contact 0 on the left and contact 5 on the right) were located in the postero-ventral part of the ALIC (see Fig. 1). The stimulation protocol was conducted exactly the same way after both surgeries. So, any parameters combination tested in the 2^nd^ surgery (including low-frequency paradigms) were also tested after the first implant, with null results.

After 16 weeks, compulsive behavior began to improve: YBOCS score fell from 35 to 26, a 32 % improvement compared with baseline (symptom severity in obsessions and compulsions decreased in parallel). A Dimensional Yale-Brown Obsessive-Compulsive Scale (DY-BOCS) was also scored after second surgery, showing that clinical improvement occurred mostly in the Aggressive-Checking dimension (Basal Severity Score: 15 vs Postsurgery Severity Score: 9, 40 % reduction) and secondly in the Symmetry-Ordering dimension (Basal Severity Score: 15 vs Postsurgery Severity Score: 10, 33 % reduction). The patient reports improved quality of life and a stabilization of daily life routines (in Table 1). Since hospital discharge (in June 2011) the patient remains stabilized with fluctuating moderate symptomatology (YBOCS 22 in April 2012, a 42 % improvement), but he has not needed further hospital admission.

## Discussion

Although there is broad consensus on the neuropathological correlates of OCD, the search for the “OCD target” for DBS is still ongoing. Various targets have been chosen with the aim of modulating activity within the cortico-striato-thalamo-cortical circuit (CSTC) at one of its critical nodes. Some of these regions are the ALIC [12], the nucleus accumbens [13], the ventral caudate nucleus [14], the VS/VC [11], the inferior thalamic peduncle (ITP) [15] and the subthalamic nucleus [16], in which differing therapeutic benefits have been achieved. Clinical outcome for all studies yields a mean Y-BOCS reduction that ranges from 45.1 to 47.7 % and a global percentage of responders of 60 % [17, 18]. Previous evidence supported that reductions in OCD severity were greater in patients receiving NAcc and VC/VS stimulation (52-54 %) than in patients receiving STN stimulation (41 %) (reviewed in [17]). However, in a recently published meta-analysis comparing the response to DBS in all available OCD patients worldwide, no significant differences were detected between targets in terms of the percentage of reduction in Y-BOCS scores or in percentage of responders when striatal areas stimulation were compared to STN stimulation [17]. These results must be taken with caution, as studies are also heterogeneous in terms of electrode design and stimulation parameters (and patients are also clinically heterogeneous).

Our patient did not respond satisfactorily to the first surgery, although the neuroanatomical target used was similar to that used in other patients enrolled in our DBS program who did respond. We decided to use a double strategy for the second surgical procedure. First, we implanted the lead in a more posterior location. Targeting a more posterior region of the VS/VC has been associated with a greater YBOCS reduction [11]. The VS/VC has been reported to be a node of different CSTC circuits related with OCD symptoms. Neuromodulation of these circuits seem to be more effective with a more posterior location of the DBS electrodes since these circuits become more compact as they run posteriorly to the thalamus through the inferior thalamic peduncle (ITP) [11]. Indeed, in our patient, stimulation seems to be centred on the anatomical region of the ITP target described by Jimenez [15].

In the second intervention we also used a larger electrode (Model 3391). This lead was designed to cover the length of the ALIC (active contact length of 3 mm). It seems plausible that, if this lead stimulates a wider range of neuroanatomical areas, the combination of parameters including the optimum target for this patient is more likely to be found with the second implant. The final position of the active contacts is in close relation with certain brain structures believed to be involved in pathophysiological aspects of OCD (Fig. 1). The bed nucleus of the stria terminalis (left side) has been related to anxiety expression in some experimental models [19], and the medial forebrain bundle (the deepest active contact on the right side) is known to be a key structure in the brain’s reward circuitry with a potential involvement in compulsive behavior [20]. Although it is speculative, this could lead us to hypothesize that different subgroups of OCD (according to phenotypic differences) could respond better depending on the target stimulated. Unfortunately, data available in literature does not allow drawing reliable conclusions on this issue due to several reasons: the number of patients included in OCD DBS studies are insufficient to allow comparisons between targets, OCD DBS studies differ in electrode design and stimulation protocols used (which also hampers the comparison between clinical issues and targets), and current magnetic resonance imaging does not have enough resolution to find differences between NAcc and VC/VS (which are very close).

The therapeutical response after the second surgery was moderate despite optimal anatomic placement, and multiple stimulation parameter settings were performed for several months (monopolar, bipolar, stimulation of multiple contacts). This particularly resistant profile observed in some patients has been ascribed to an individual response to DBS, according to differential clinical features and patterns of connectivity (reviewed in Lipsman) [21]. It has been reported that ‘just-right’ experiences or the need for symmetry exhibited by our patient may be less likely to respond to DBS [22, 23]. Moreover, early onset OCD has been associated to a worse response to DBS when all targets are considered as a whole [17]. Regarding the existence of differential patterns of connectivity, the use of neuroimaging techniques such as diffusion tensor imaging (DTI)-based tractography has been proposed to identify the precise trajectory of tracts at a single subject level in depression [24].

The patient described became a responder after reimplantation of DBS electrodes in the VS/VC target in a more posterior location. The stimulation of different brain areas after an unsuccessful implantation has been previously described in Tourette syndrome patients with severe comorbid OCD (adding bilateral ALIC/NAcc region DBS leads to the existing Vo/CM-Pf stimulation or through de novo ALIC/NAcc DBS plus Vo/CM-Pf region stimulation) [25]. However, the explantation and reimplantation of DBS devices has not been previously reported in the literature for therapeutical benefits in resistant OCD.

The experience of our case brings us back to the debate on which is the best target for OCD, as the stimulation of both targets (NAcc and VC/VS) have been reported to be effective in resistant patients. They are very close and one could think that the stimulation of one would influence the activity of the other, as they are critical nodes within the CSTC circuit. Evidence in the literature does not allow us to know why our patient did not benefit from the first implantation in NAcc (although he did from the second implantation, in VC/VS) nor why he responded while receiving low frequency DBS. Although it is speculative, it cannot be discarded that some targets might be more sensitive to electric stimulation than others (due to still unknown mechanisms). It would be similar to the fact that some patients respond to certain selective serotonin reuptake inhibitors (SSRIs), but not to others (when all drugs belong to the same family and theoretically share the same mechanism of action). The target selection is further complicated, as it has been demonstrated with neuroimaging data that even within the same NAcc, the actual stimulation coordinates vary substantially between patients, which significantly influence on clinical outcomes [26]. So, depending on the specific stimulation site, different fiber bundles and therefore different sets of structures may be affected [11]. From an electrophysiological point of view, stimulation both hyperpolarize and depolarize neurons and activation of axons may have distal effects, including overall excitation or inhibition through stimulation of inhibitory neurons. This indicates that DBS may evoke multiple effects (reviewed in [27]). The benefits of low-frequency DBS has been reported in animal models of OCD. The 8-OHDPAT-induced decreased alternation model might serve to model two specific aspects of OCD, namely perseveration and indecision (reviewed in [17]). In this rat model, low- but not high-frequency stimulation (HFS) of the thalamic nucleus is effective in reducing 8-OHDPAT-induced perseveration [28] whereas HFS of the STN has shown anti-OCD effects in humans [29]. Future research will help to integrate findings coming from different research approaches.

Some weaknesses might be considered: 1) potential effects of other concurrent interventions performed when the patient responded (besides the explantation and the stimulation of a new target) cannot be discarded. This interventions include slight changes in medication, change in type of electrodes, changes in stimulation parameters and possibly a change in volume of tissue activated (VTA) post surgery 1 vs post surgery 2. Unfortunately, we are unable to ensure in which extent these interventions were involved in the clinical benefit. We think that VTA in second surgery represented a smaller contribution in clinical improvement. Although we cannot estimate the VTA directly, we have calculated charge densities in first and second surgeries (this parameter is proportional to VTA), resulting that it was about half in the second surgery, compared to the first surgery (data to perform the calculation are available at the Table 1). On the other hand, although some reports suggest that DBS may allow previously ineffective pharmacological treatments to become effective [30], it is difficult for the authors to assume that the rise of the dose of an SSRI drug may be responsible for the improvement of a patient who did not respond to very potent drugs as phenelzine or clomipramine at full doses; 2) On the other hand, a morphing of the atlas to the patient’s own landmarks was not performed to determine anatomic localization of the target. However, the authors believe that this did not affect significantly to the accuracy of the final positioning of leads.

## Conclusions

Our report emphasizes the reversibility profile of DBS, and helps to dissociate it from the historical negative connotations of psychosurgery. Following up a patient with persisting disabling symptoms and very poor quality of life implies an ethical and clinical dilemma, especially when the alternative of a second surgery is not exempt from risks. Although the experience of a case cannot be extrapolated to another, the authors believe that a second surgery could be considered after 18 months of non-response to the first surgery in OCD resistant patients (when all programming possibilities have been exhausted). The capability for explantation and reimplantation of DBS leads should be considered as part of DBS reversibility profile in resistant mental disorders, as it allows application in cases of non-response to the first surgery.

## Consent

Written informed consent was obtained from the patient for publication of this Case report and any accompanying images. A copy of the written consent is available for review by the Editor of this journal.
